# Machine learning algorithms to forecast air quality: a survey

**DOI:** 10.1007/s10462-023-10424-4

**Published:** 2023-02-16

**Authors:** Manuel Méndez, Mercedes G. Merayo, Manuel Núñez

**Affiliations:** Design and Testing of Reliable Systems Research Group, Universidad Complutense de Madrid, C/ Profesor José García Santesmases, 9, 28040 Madrid, Madrid Spain

**Keywords:** Machine learning, Deep learning, Regression algorithms, Air quality

## Abstract

Air pollution is a risk factor for many diseases that can lead to death. Therefore, it is important to develop forecasting mechanisms that can be used by the authorities, so that they can anticipate measures when high concentrations of certain pollutants are expected in the near future. Machine Learning models, in particular, Deep Learning models, have been widely used to forecast air quality. In this paper we present a comprehensive review of the main contributions in the field during the period 2011–2021. We have searched the main scientific publications databases and, after a careful selection, we have considered a total of 155 papers. The papers are classified in terms of geographical distribution, predicted values, predictor variables, evaluation metrics and Machine Learning model.

## Introduction

The economical and urban development of cities has brought that the interest in environmental pollution has risen during the last years. Among other problems, air pollution has a big impact in human health, being a risk factor for many diseases that can lead to death. According to the World Health Organisation (WHO), air pollution is a “silent killer” that produces the premature death of almost seven million people each year,^1^ including 600.000 children.

Air pollution forecasting is very useful for informing about the pollution level that will allow policy-makers to adopt measures for reducing its impact. For example, traffic restrictions could be imposed with the goal of avoiding high pollution episodes. Usually, the Air Quality Index (AQI) is used to indicate the air pollution level. AQI is a piece-wise linear function of the following pollutant concentrations: Ozone ($O_{3}$), particulate matters (*PM*2.5, *PM*10), carbon monoxide (*CO*), sulphur dioxide ($SO_{2}$) and nitrogen dioxide ($NO_{2}$). Nevertheless, there does not exist a global standard and different countries and regions have their own AQI indexes, based on their own air quality standards.

Machine Learning (ML) techniques are the most common methods to forecast air quality. Since the beginning of the 21st century, we can find hundreds of works in the literature that propose implementations of different models to get the best accuracy in the forecasting of air quality index or pollutant concentration prediction.

There has been much research on applying different ML algorithms to predict both the AQI and the level of concentration of specific pollutants related to air quality. In fact, there are some papers that collect applications of ML to air quality (e.g. Niharika Venkatadri and Rao 2014; Nemade 2019; Iskandaryan et al. 2020; Bharat Deshmukh et al. 2021; Sharma et al. 2021). Unfortunately, their scope is rather limited, the total number of analysed papers is very small and the considered classification categories are very restricted. Therefore, we are not aware of an extensive work that reviews this important field.

The thorough study of these papers provide us the followings insights:

1. The pollution and technological potential in countries are determinant factors to publish papers in this topic.
2. From 2011, with few exceptions, there is an increase on the number of papers per year in the topic. This trend is very clear between 2017 and 2020. However, this trend (surprisingly) changed in 2021. We concluded that this change was due to the emergence of COVID-19 data as main case study of research working on time series forecasting. In order to support this claim, we did a simple experiment: we queried *IEEE Xplore* using *’("Abstract":"air quality") AND ("Abstract":"machine learning" OR "Abstract":"neural network" OR "Abstract":"prediction" OR "Abstract":"regression")’* and obtained a total of 105 results in 2020. Then, we modified the query and the year: we replaced “air quality” by “COVID” and checked 2021. We obtained a total of 1108 results.
3. AQI is the most predicted pollutant measure and *PM*2.5 is the most predicted pollutant concentration.
4. The most employed predictor variables are pollutant features ($50\%$) followed by weather features ($35\%$) and the combination of both of them gives the best accuracy.
5. Evidence seems to point towards deep learning algorithms being more effective than regression algorithms.
6. Despite the rise in popularity of deep learning, the ratio of deep learning algorithms with respect to regression algorithms has remained almost constant over the years.

The rest of the paper is organised as follows. In Sect. 2, we describe the main ML algorithms that have been applied for prediction of air quality. In Sect. 3 we explain the review methodology that we have applied and how we select publications by taking into account different aspects. Specifically, we consider studied geographical area, predicted values, predictor variables and evaluation metrics. In Sect. 4, which constitutes the bulk of the paper, we describe the papers that we have included in this survey according to the underlying algoritm. Finally, in Sect. 5 we present our conclusions.

## Machine learning and classical regression algorithms

Machine Learning is a branch of Artificial Intelligence that aims to provide computers with the ability to learn how to perform specific tasks without being explicitly programmed by a human. This technique is based on the design of models that learn from data and make decisions or predictions when new data are available. Deep Learning (DL) can be seen as an evolution of ML that uses a structure of multiple layers called Artificial Neural Network (ANN). DL algorithms require less involvement of humans because features are automatically extracted. However, an important difference with respect to other ML techniques is that DL requires massive data to work properly.

Although ML and DL are recent concepts, the first computer learning program was written by Arthur Samuel in 1952 and the first neural network was proposed by Frank Rossenblatt in 1957.^2^ Since the 1990s, the development in both ML and DL has been significant, mainly due to the increment of computation power and the availability of large amounts of data.

There exist many ML approaches that can be applied to solve different problems. In this section, we will review only those algorithms that have been used for predicting pollutant measures. We can distinguish between the ones based on regression analysis and the ones using neural networks. Moreover, in the first category we will distinguish between the use of classical regression algorithms and ML algorithms.

### Classical regression-based algorithms

Regression analysis is used to infer the relation between a dependent variable and a set of independent variables. On the basis of this relation, and using the values of the independent variables, the value of the dependent variable is estimated. Regression helps to predict a continuous value. Next we review classical algorithms to carry out regression.

- **Multiple Linear Regression (MLR).** Let *y* and $x_{1}, \ldots , x_{p}$ be the dependent variable and the independent variables, respectively. The goal of linear regression is to define a linear function $f(x_{1}, \ldots , x_{p})$ that minimise the square mean error, that is,
  $$\begin{aligned} min[(y - f(x_{1}, \ldots , x_{p}))^2] \end{aligned}$$
  If $p = 1$ the algorithm is called *simple linear regression*.
- **Auto-Regressive Integrated Moving Average (ARIMA).** This model uses time series for forecasting, that is, it makes predictions based on past and present data. The ARIMA model includes three components. The auto-regressive component refers to the number of delays used in the model. For example, an auto-regressive value of 3 establishes that only the three previous values will be used to explain the current value. The integrated component represents the differentiation degree required to convert the time series into a stationary series. The last component, the moving average, refers to the number of past errors required to explain the current error. Again, if the value of the moving average is 3, then only the three previous error values can be used to explain the current error value. When the time series is seasonally, it uses an ARIMA variant called SARIMA.

### Machine learning regression-based algorithms

In this section we review ML algorithms used for air quality prediction based on regression analysis.

**Fig. 1:**
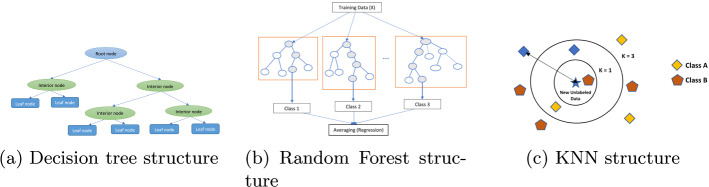
Graphical representation of ML regression-based algorithms

- **Support vector regression (SVR).** Support vector machines are mainly used in classification problems. However, they can be also applied to regression. In this case, the approach is called support vector regression. Let *y* and $x_{1}, \ldots , x_{p}$ be the dependent variable and the independent variables, respectively. Basically, SVR works as follows. First, a linear regression function, that is, a hyper-plane $h(x) = w_{1} x_{1} + \ldots + w_{p} x_{p} + b$, must be defined. Then, a margin of tolerance $\varepsilon$ is considered, expecting that all data will be at most at distance $\varepsilon$ from the hyper-plane. In case the deviation of some points is larger than this value, *slack variables* $\xi , \xi '\ge 0$ can be introduced to cope with them. The final goal is to find the minimum of the function:
  $$\begin{aligned} \min \left( \frac{1}{2} ||w||^2 + C \sum _{i = 1}^{N} (\xi + \xi ')\right) \end{aligned}$$
  considering the restrictions
  $$\begin{aligned} y - w\cdot x_{i} - b \le \varepsilon + \xi \\ w\cdot x_{i} + b - y_{i} \le \varepsilon + \xi ' \end{aligned}$$
  In case of non-linear functions, SVR uses kernel functions to transform data into a higher dimensional space in order to develop a linear regression transformation.
- **Decision trees (DT).** The aim of this algorithm is to design a model for predicting a quantitative variable from a set of independent variables. The algorithm is based on a recursive partitioning. Trees are composed of decision nodes and leaves. DT regression usually is built by considering the standard deviation reduction to determine how to split a node in two or more branches. The root node is the first decision node that is divided on the basis of the most relevant independent variable. Nodes are split again by considering the variable with the less sum of squared estimate of errors (SSE) as the decision node. The dataset is divided based on the values of the selected variable. The process finishes when a previously established termination criterion is satisfied. The last nodes are known as leave nodes and provide the dependent variable prediction. This value corresponds to the mean of the values associated to leaves. Figure 1a Balogun and Tella (2022) shows a graphical representation of the general structure of a standard Decision Tree.
- **Random Forest (RF).** Random Forest is based on the generation of several decision trees. The prediction will be the average of the predictions provided by the different trees. For the construction of each decision tree, a data sample is selected from the training dataset. The rest of the data will be used to estimate the decision tree error. The subset of independent variables that can be used for splitting each node are randomly selected. *Extremely randomised trees* (ERT) is a slightly modified random forest algorithm. Figure 1b Balogun and Tella (2022) shows a graphical representation of the general structure of a Random Forest Regressor.
- **K-nearest neighbours regression (KNN).** The *k*-nearest neighbours algorithm is often applied to classification problems although it can be also applied to regression problems. The idea of this algorithm is simple. Given a distance (Euclidean distance, Mahalanobis distance, etc) and a *k* value, the algorithm calculates the distance between a data point and the training dataset points for selecting the *k* nearest ones and establishes the average of them as prediction. An improvement of this algorithm is the algorithm known as *weighted* *k*-*nearest neighbours* (WKNN). In this case, the prediction considers a weighted arithmetic mean for calculating the prediction. Figure 1c Tella et al. (2021) shows a graphical representation of the general structure of a KNN model.

### Deep learning algorithms

DL algorithms use ANNs. In this section, we will briefly describe different types of ANNs used in the literature for air quality prediction. In order to understand the internal behaviour of an ANN, we will introduce its structure.

An ANN is an algorithm based on the biological neuronal connections that are comprised of neurons or nodes. These connections are organised in three layer types. The input layer receives as input the original predictor variables. The output layer produces the predicted value for the given inputs. These two layers are connected by the the hidden layers. The hidden layers (more than one in the case of deep learning) contain non-observable neurons which are in charge of the computation. Each node in a layer is connected with nodes in the next layer. Each connection has associated a weight that is used to combine the inputs. Each node or neuron in the next layer receives the weighted value and transforms it by means of an activation function. Sigmoid and rectified linear unit functions are the most popular. The obtained result is the value that is passed as input to the nodes in the next layer. This process continues until the output layer is reached. At this point, the output prediction is produced.

The final aim of an ANN is to fit the weights to minimise an error function, commonly a quadratic function. To do this, the ANN uses the known as back-propagation algorithm. This algorithm employees the gradient descendent method using the layers partial derivatives to find the optimal weight of each node.

Now, we describe the different types of ANNs that have been used for predicting pollutant measures.

- **Multi-layer perceptron neural networks (MLP).** MLPs are classical neural networks with one or multiple hidden layers.
- **Convolutional neural networks (CNN).** CNNs are usually applied to images. They are an extension of MLP in which two types of layers are alternated: convolutional and pooling layers. The goal of convolutional layers is to extract features from the input image. A convolutional operation, which outputs convoluted features, is carried out by a matrix called kernel or filter. These features are the input of pooling layers. The aim of a pooling layer is to reduce the size of the convoluted features with the goal of decreasing the computational power required to process the image.
- **Recurrent neural networks (RNN).** RNNs deal with either time series or sequential data, that is, information ordered and related. RNNs have an internal memory in the sense that a neuron can feedback itself by receiving as input the output it has previously produced. This allows the model to acquire short-term memory which is essential to time series forecasting.
- **Long-short term memory neural networks (LSTM).** LSTMs are an extension of RNNs. They have an extended memory that allows to deal with long-term dependencies. LSTMs can remember information over arbitrary time intervals. The core component is the cell state that carries information throughout the processing of the data. The information is updated on the basis of three gates. Each of them controls the information that should be in the cell state using a sigmoid activation function. The forget gate determines which part of the previous state information should be forgotten. The input gate decides the new information that will be used to update the memory. Using an hyperbolic-tangent function, it creates a vector candidate to be added to the cell state. The last gate, known as output gate, uses a *tanh* activation function for determining which part of the updated cell state will be used as output.
- **Gated recurrent unit (GRU).** GRUs are a simplified version of LSTMs that combine the forget and the input gates. They provide similar results to the ones obtained by LSTMs.
- **Encoder-Decoder neural networks (EDNN).** The encoder-decoder model is a recurrent neural network used for sequence-to-sequence prediction problems. Its architecture consists of three components: encoder, intermediate vector and decoder. The encoder and the decoder are comprised of a set of recurrent units (usually, LSTM or GRU). In the case of the encoder, each unit processes an element of the input sequence and tries to encapsulate the associated information in the intermediate vector, for increasing the accuracy of the decoder prediction. The decoder uses this vector for producing the prediction.

## Review methodology

In order to collect the papers included in this survey, we searched the main online research databases of technical publishers. Specifically, we considered ACM Library, Elsevier Online Library, IEEEXplore, SpringerLink and Wiley Online Library. We based the search on the occurrence of some keywords on the abstracts of the publications, with the exception of SpringerLink. In this case, it was not possible to restrict the exploration to the abstract section, so we search in the title of the papers. The usage keywords were “machine learning” “neural network", “regression" and “prediction", each of them combined with “air quality". We only collected papers published between 2011 and 2021. The total number of papers obtained from all considered databases was 781. Next, we examined the papers more deeply to select only those papers where air quality prediction was the main topic.

**Fig. 2:**
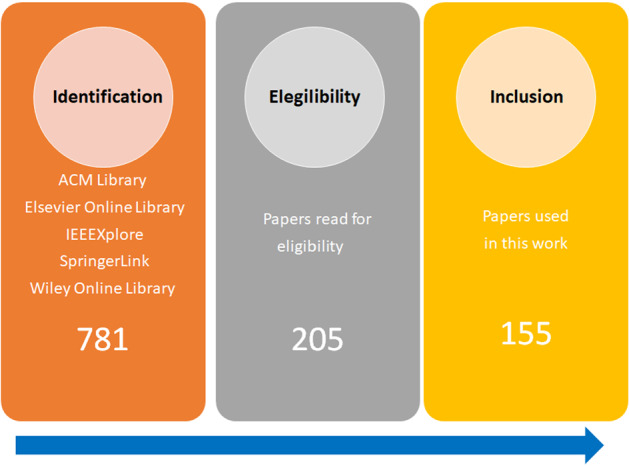
Flow chart for paper identification and selection

First, we removed duplicated papers and papers not written in English. In this phase, we also examined the publications in order to identified those ones that did not fall in the scope of the survey, that is, the application/development of ML algorithms to predict future values of AQI or pollutant concentrations. After filtering these papers, we obtained a set of 205 publications. Next, we went in depth in the reading of the papers for which it was not very clear whether they dealt with air quality prediction. In particular, we checked papers that did not make predictions about the future, but only analysed data to determine current AQI values. Finally, the total number of papers was reduced to 155. In Fig. 2, following the structure presented on a recent survey on the inter-correlation of climate change, air pollution and urban sustainability using novel machine learning algorithms and spatial information science Balogun et al. (2021), we present our flow chart for paper selection, screening, eligibility and inclusion.

**Fig. 3:**
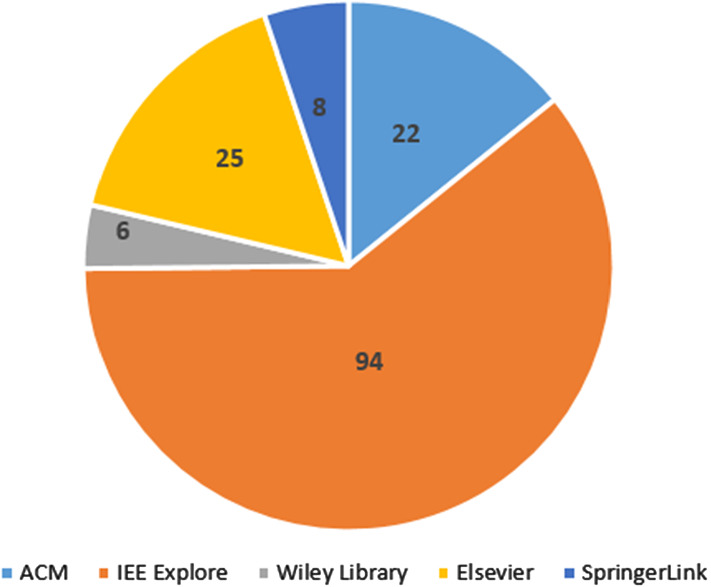
Selected papers by database

**Fig. 4:**
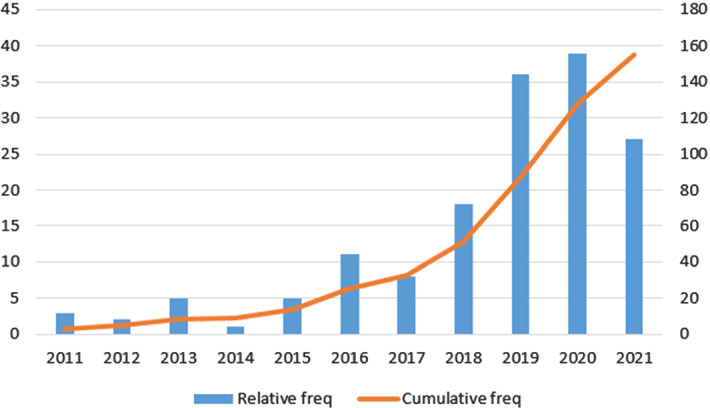
Papers by publication year

In Figs. 3 and 4 we present the distribution of the final papers based on the search engines from which they were obtained and on the year of publication.

Next we analyse how we will classify publications in terms of geographical distribution, predicted values, predictor variables used for forecasting and evaluation metrics used to determine the models quality.

**Fig. 5:**
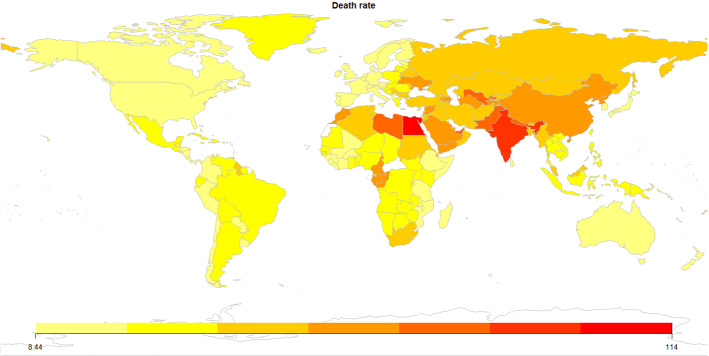
Death per 100.000 population caused by air pollution in 2017

**Fig. 6:**
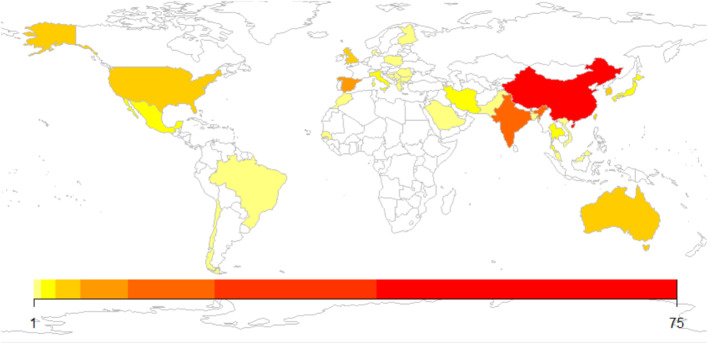
Number of papers by country in the survey

### Geographical areas

Air pollutant emissions strongly vary among different countries. Usually, developing countries are more polluted than developed countries. Specifically, East and South Asia are the most affected regions. According to *Our world in data*^3^, in 2017, East Asia ($9.75\%$), South Asia (7.63%) and Central Asia (6.19%) are the world regions that present the highest share of deaths by air pollution. In particular, China and India are the countries with more deaths due to this cause, 1.030.000 and 819.000, respectively, in absolute terms. In relative terms, Egypt (114.2 deaths per 100.000) and India (88 deaths per 100.000) are the most affected countries. As we can see in Fig. 5, Middle East and North Africa are also regions that suffer the air pollution impact in terms of human lives. In regions such as North Europe, North America, Australia and New Zealand, the deaths caused by pollution are much lower than in the aforementioned regions.

Figure 6 illustrates how the specific country influences the number of publications related to the application of ML techniques for prediction of air pollution. The most influential advances in the field of ML applied to air quality prediction have been made by researchers in China. This fact is due to the scientific and technological potential of Chinese universities and the big air pollution problem they suffer. In this survey, more than half of the considered papers apply some ML technique to the air quality forecast of a Chinese city. The air quality of cities such as Beijing, Shanghai, Hangzhou and Guangdong has been analysed and predicted in many recent approaches using different ML algorithms (e.g. Ma et al. (2019); Yang et al. (2020); Lan et al. (2020); Han et al. (2021) among many others).

As we said before, India is also a country really harmed by air pollution, even more than China. According to the *IQ Air*,^4^ nine of the top ten polluted cities in the world are Indian. Unfortunately, scientific and technological resources in India are lower than the Chinese ones and this is probably the main reason why papers that apply ML techniques to predict either air quality or pollutant indexes in Indian cities is very low. Nevertheless, many of the publications that compare different models to forecast air quality use data from Delhi. We can also see that in Europe and America, ML is not so widely used as in Asia for predicting air pollution. In Europe, Spain is the country in which most papers have been produced (7) followed by United Kingdom (6). Further on, one can see that in this survey there are papers studying air quality of many countries in East Europe. It may be because this is the European region most affected by pollution. In the rest of the world, Australia (6) and USA (4) lead the way.

### Predicted values

AQI is used by institutions to provide the population with information about the pollution degree of cities or neighbourhoods taking into account the main pollutant gasses. Therefore, it is natural that AQI is the most studied magnitude for being predicted in order to determine the air quality. It is worth pointing out that different countries use different AQI scales. We can mention the following four indexes.

- The Common Air Quality Index,^5^ used in Europe since 2006, considers 5 risk categories and ranges from 0 (low risk) to 100 (air quality is unhealthy).
- The China Air Quality Index^6^ has 6 risk categories and ranges from 0 (excellent) to 500 (severed polluted).
- The US Air Quality Index^7^ runs from 0 to 500. These values are divided into 6 categories that correspond to different levels of air quality.
- In the case of United Kingdom,^8^ it runs from 0 to 10, classifying these values into 4 levels.

**Fig. 7:**
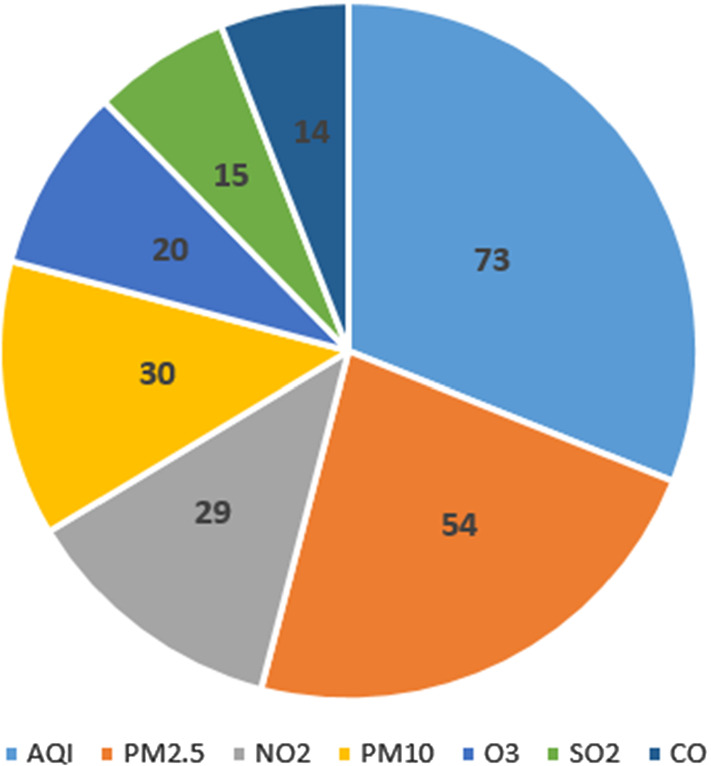
Publications by predicted pollutants

There exist several pollutants that are also considered for their prediction: carbon monoxide(*CO*), ozone ($O_3$), sulphur dioxide ($SO_2$), nitrogen dioxide ($NO_2$), nitrogen oxides ($NO_X$) and particulates matter (*PM*2.5, *PM*10). However, we are not aware of a categorisation that clearly shows the harmfulness of each pollutant. The only data that could be used for analysing the level of danger of these pollutants is the one related to the premature deaths caused by each of them. Unfortunately, due to the difficulty in determining this fact, it is not easy to find this information. We just found data of premature deaths attributable to *PM*2.5, $NO_2$ and $O_3$ from the European Environment Agency.^9^ According to this source, in 2018, *PM*2.5, $NO_2$ and $O_3$ were the cause of 379.000, 54.000 and 19.000, respectively, premature deaths in the European Union. There are no data for other pollutants. According to the World Health Organisation,^10^*PM*10 are unhealthy for human, but no so much as *PM*2.5 because of its size. In the case of *CO*, according to the UK Committee on the Medical Effects of Air Pollutants,^11^ during the last years there has been an appreciable reduction of outdoor concentrations which makes that its danger has also decreased. These considerations help us to justify the correlation between the danger of the pollutants and the volume of papers devoted to forecasting them. Figure 7 shows the number of analysed publications in this survey for the prediction of each of the pollutants. Note that several papers predict more than one pollutant.

### Predictor variables

In this Section we will analyse the independent and predictor variables that are used by models for pollutant forecasting. We have only considered those variables that are used by at least $5\%$ of the models developed in the analysed publications. We distinguish three types of variables.

- *Pollutant variables* correspond to pollutant concentrations.
- *Weather variables* are associated with different weather elements: wind direction, wind speed, atmospheric pressure, rain, relative humidity, temperature and dew point.
- *Other variables* include different data such as year, season, month, week, weekday, hour, geolocation and visibility.

Note that several papers included in this survey use two or more dependent variables.

Next, for each of these categories, we enumerate the percentage of analysed publications that use them as independent variables for predicting AQI, *CO*, $O_3$, $SO_2$, $NO_2$, *PM*2.5 and *PM*10.

**Fig. 8:**
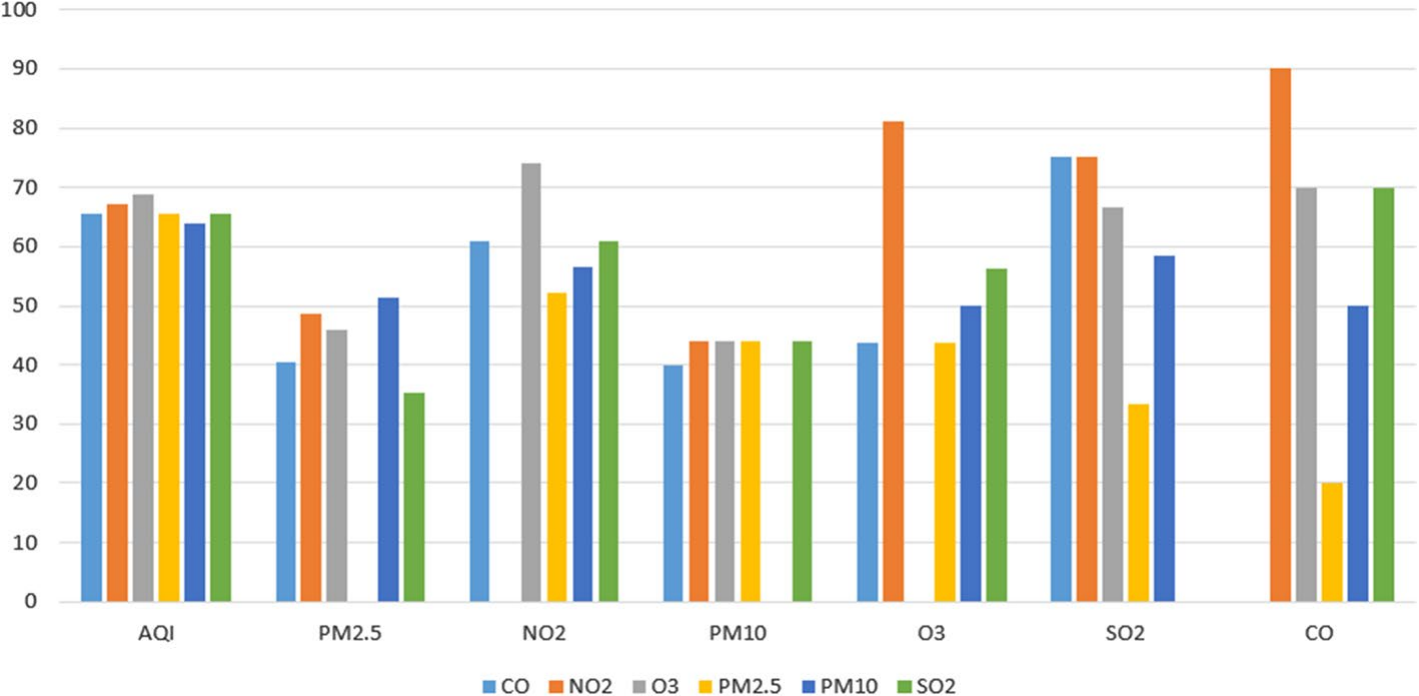
Pollutant variables by dependent variable (percentage)

Figure 8 shows the relevance of pollutant variables for predicting the different dependent variables. As can be noted, all the predictor variables are used in a similar percentage in the design of the analysed models. It is not surprising that all the pollutant variables are used in equivalent percentages for AQI prediction. This is due to the fact that all the pollutants have the same relevance in the piece-wise linear function to estimate the AQI value. However, there exist differences in the case of the prediction of other pollutants. It is remarkable the influence of $NO_{2}$ at the time of predicting *CO*. $NO_{2}$ is also the most important pollutant feature when other pollutants are predicted, like *CO*, $O_{3}$ or *PM*10. On the contrary, *PM*2.5 is the pollutant variable less used in the design of models for predicting other pollutant concentrations. The rest of pollutant variables are used in a similar percentage.

**Fig. 9:**
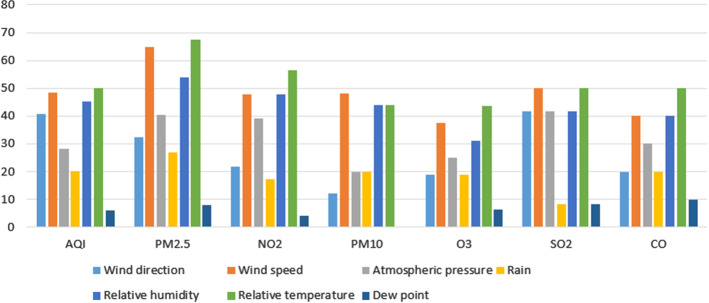
Weather variables by dependent variable (percentage)

Figure 9 presents the use of weather variables as predictors in AQI and pollutant forecasting. The first thing that can be observed is that although all of them are considered to some extent in the design of the models, their presence in the different proposals is not as important as that of the pollutant variables. The relative temperature, the relative humidity and the wind speed are the weather predictors most included in the models (used in around a $50\%$ of them). In a lower percentage we find the atmospheric pressure, the wind direction and the rainfall (used in around a $35\%$ of the models). We can also detect a significant difference between the use of wind speed and wind direction in the prediction of all the pollutants: the latter is more widely used than the former. The less used variable of this type is the dew point, (used by less than $10\%$ of the models). We can also observe that the dew point is not taken into account for predicting *PM*10.

**Fig. 10:**
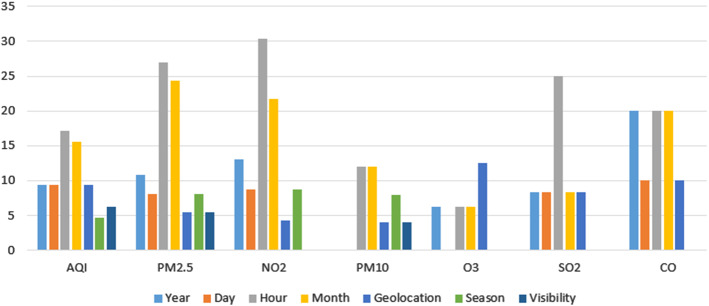
Other variables by dependent variable (percentage)

Figure 10 shows the influence of other variables in the proposed models. In this category, the different approaches included in this survey mainly consider variables related to the timeline such as hour, day, month, weekend day and year. In addition, we also analysed the impact of the geographical location, that is, the geolocation and the visibility. Although other features appear in some models, like illumination, snow, land use and solar radiation, among others, as we previously said, we have only considered the variables used by at least $5\%$ of the proposals. The predictor variables included in this category are not uniformly distributed in the models for the prediction of the different pollutants. In general, the most frequent variables are month and hour (used in around $25\%$ of the models). These predictor variables are really significant in the predictions as external factors. In the case of the hour, the nighttime, for example, usually is associated with a reduction of the traffic and the industrial activity. Consequently, it should imply a decrease in the pollutant concentrations. In the case of the month, something similar happens. For instance, in many cities the work activity during summer is notably lower than during the rest of year. Therefore, a decrease in pollutant concentrations can be expected. On a second level we find geolocation, day and year (used in around $15\%$ of the models). Finally, season and visibility are the less used variables of this category. In particular, season is not used in any proposed model for the prediction of *CO*, $SO_{2}$ and $O_{3}$. $O_{3}$ predictor models do not use day and *PM*10 predictor models do not use year. On the contrary, hour is the most used variable of this type in all the predictions except for predicting $O_{3}$.

**Fig. 11:**
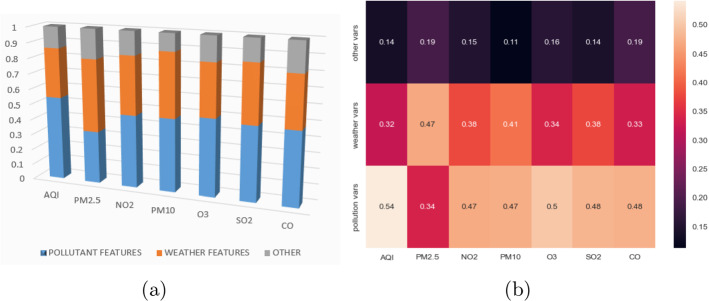
Use of predictor variables by dependent variable

Figure 11 illustrates the influence of the different types of variables in the prediction of the different pollutants. In all the cases, except *PM*2.5, the pollutant variables are the most used (around $45\%$), followed by the weather variables (around $35\%$). The rest of predictor variables are only used by around $15\%$ of the models. However, in the case of *PM*2.5 the weather variables ($50\%$) have an essential role being more used than the pollutants variables ($33\%$).

### Evaluation metrics

Evaluation metrics help us to determine the quality of an ML model. We classify them in two groups. On the one hand, range-dependent metrics compare different models that must be applied to the same dataset. On the other hand, percentage metrics compare models independently of the dataset used.

The range-dependent metrics used in the analysed publications are described in Table 1, where $Y_{i}$ and ${\hat{Y}}_{i}$ correspond to the current and the predicted values of the *ith* observation, respectively, and *n* represents the number of observations. In Table 2 we can see the percentage metrics, where ${\bar{Y}}$ indicates the mean of the observations, *TP* the number of true positives, *FN* the number of false negatives and *FP* the number of false positives.

**Table 1:** Range-dependent metrics

MSE	Mean square error	$\frac{1}{n} \sum _{i = 1}^{n} (Y_{i} - {\hat{Y}}_{i})^2$
RMSE	Root mean square error	$\sqrt{\frac{1}{n} \sum _{i = 1}^{n} (Y_{i} - {\hat{Y}}_{i})^2}$
MAE	Mean absolute error	$\frac{1}{n} \sum _{i=1}^{n} \vert Y_{i} - {\hat{Y}}_{i} \vert$
MRE	Mean relative error	$\frac{1}{n} \sum _{i=1}^{n} \vert \frac{Y_{i} - {\hat{Y}}_{i}}{Y_{i}}\vert$

**Table 2:** Percentage metrics

MAPE	Mean absolute percentage error	$\frac{100}{n} \sum _{i = 1}^{n} \vert \frac{Y_{i} - {\hat{Y}}_{i}}{Y_{i}}\vert$
ACC	Accuracy	$1 - \frac{\sum _{i=1}^{n} \vert {\hat{Y}}_{i} - Y_{i}\vert }{\sum _{i = 1}^{n} Y_{i}}$
$R^2$	R squared	$\sqrt{\frac{\sum _{i = 1}^{n} ({\hat{Y}}_{i} - {\bar{Y}})^2}{\sum _{i = 1}^{n} (Y_{i} - {\bar{Y}})^2 }}$
IA	Index of agreement	$1 - \frac{\sum _{i = 1}^{n} (Y_{i} - {\hat{Y}}_{i})^2}{\sum _{i=1}^{n} (\vert Y_{i} - {\hat{Y}}_{i}\vert + \vert {\hat{Y}}_{i} - Y_{i}\vert )^2}$
RECALL	Sensitivity	$\frac{TP}{TP + FN}$
PREC	Positive predictive value	$\frac{TP}{TP + FP}$
FM	F measure	$\frac{PREC \cdot RECALL}{PREC + RECALL}$

**Fig. 12:**
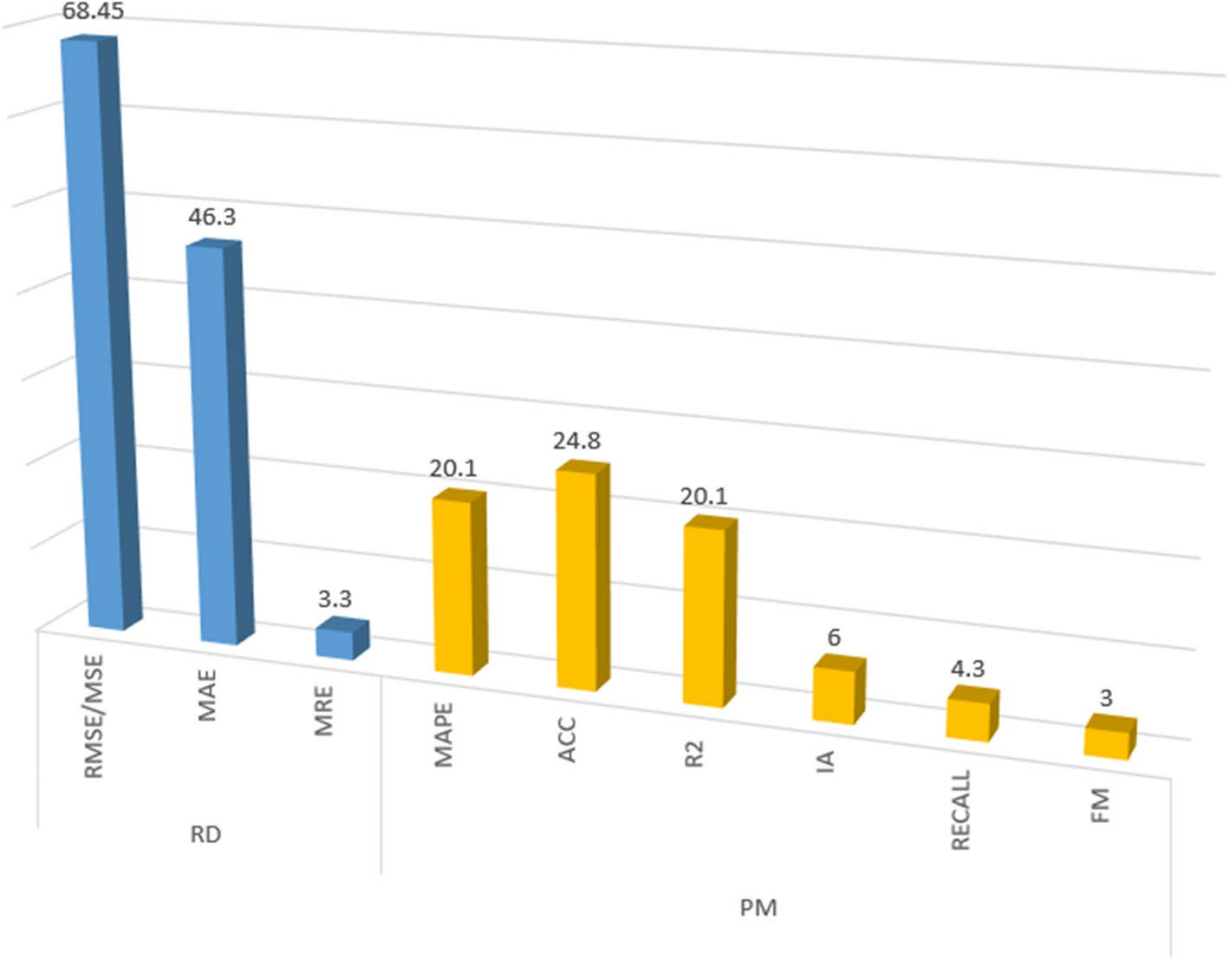
Use of evaluation metrics

As we can see in Fig. 12, the most applied metrics in the analysed proposals are ACC ($24.8\%$ of the publications), $R^2$ and MAPE ($20.1\%$ each of them). Regarding range-dependent metrics, RMSE/MSE and MAE are the most used ($68.45\%$ and $46.3\%$ of the publications, respectively). Nevertheless, as we previously said, this type of metrics does not allow comparisons between models.

The fact that pollutant variables are the most used ones is related to the strong influence on the predicted variable. Weather variables are less used than pollutant ones. Nevertheless, all but *dew point* are frequently considered. This is not surprising because meteorology is highly related to air quality. For example, in the case of ozone it is important to take into account that it is formed in the presence of sunlight. Finally, other variables related to date and geolocation have influence on the predicted variable but it is not significant. Therefore, we think that the presence of a dependent variable is directly related to its influence in the variation of the pollutant concentrations and air quality index.

## Classification, by used model, of the contributions on air quality forecasting

In this section we summarise the main contributions concerning the application of ML and classical regression algorithms to the design of air quality forecasting models.

**Fig. 13:**
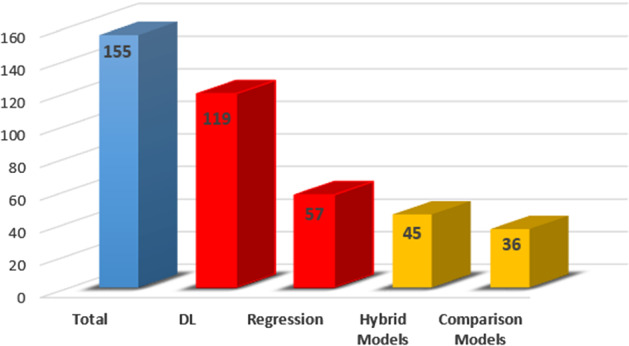
Papers by algorithm type

It is worth pointing out that some of the publications that have been studied consider several algorithms in the proposed approaches. This happens in both publications that *compare* different models and those papers in which a *hybrid model* is proposed. A hybrid model uses several algorithms to improve the performance with respect to the use of a single algorithm. In some cases, these proposals combine both deep learning and regression algorithms. Therefore, they could be classified in both types. Taking this fact into account, Fig. 13 shows the amount of publications corresponding to the different categories. In our study, we have analysed 35 publications where comparisons between approaches are studied ($22.5\%$) and 45 papers which present hybrid models ($29\%$).

**Fig. 14:**
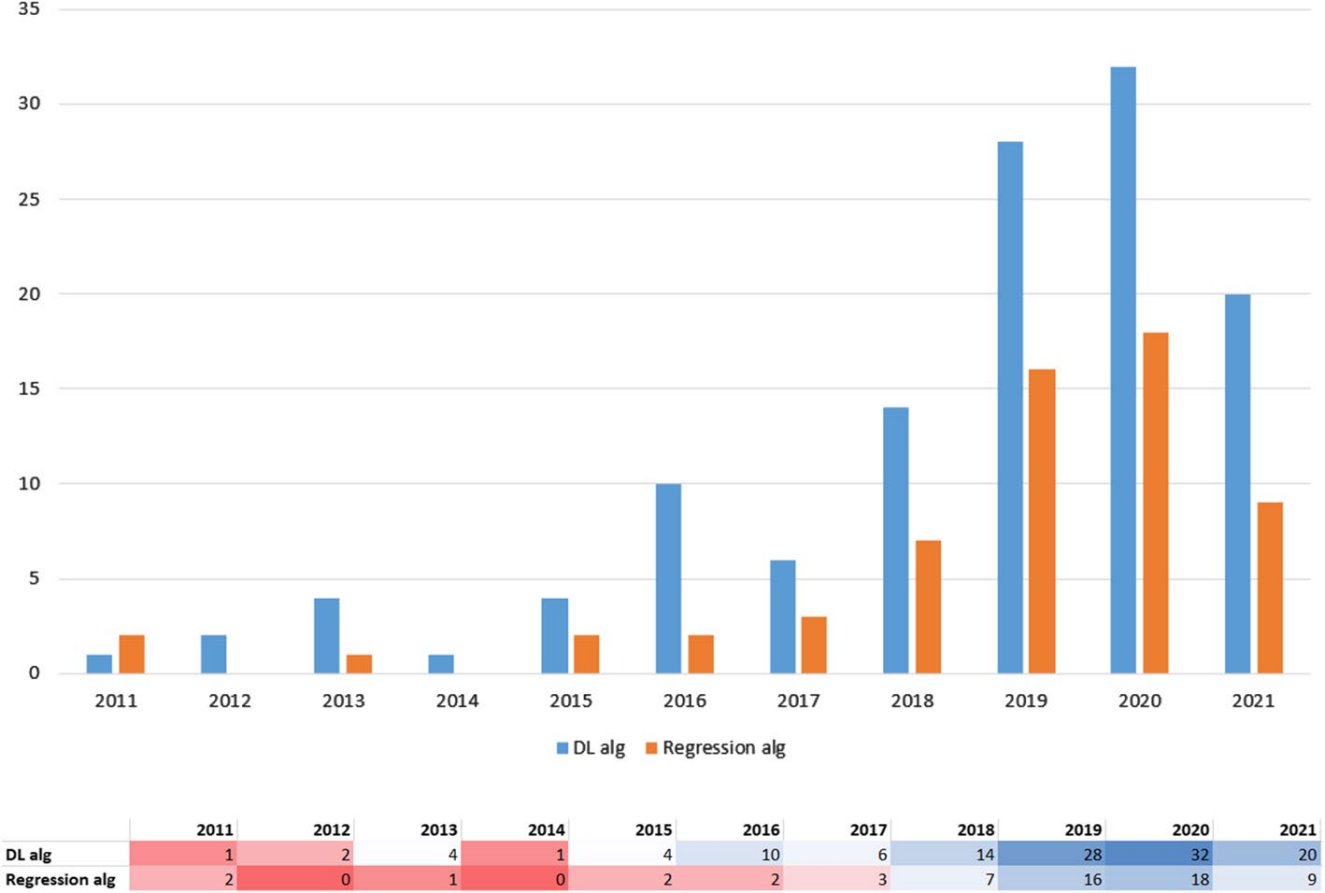
Algorithm type used by year of publication

We can also notice that the use of DL algorithms in the design of the models is most frequent than the use of regression algorithms. A total of 119 papers deal with DL methods, which represents $76.8\%$ of all the publications. Regression algorithms are considered only in 57 papers, that is, $36.8\%$ of the total number of them. This difference may be due to the “machine independence” advance of DL methods with respect to the classical regression algorithms. It makes these algorithms fit better than regression algorithms in the case of air quality forecasting because it requires the use of many predictor variables whose distribution and correlation with the target variable are not regular. Figure 14 presents the evolution of the use of both types of algorithms during the last 10 years. The publications that consider algorithms corresponding to both deep learning and regression have been included in both categories. Despite the number of publications corresponding to each of them, the increase of proposals during the last 3 years has been similar in both cases.

### Deep learning algorithms

In this Section we consider those papers that apply a single DL algorithm. Approaches that combine different DL methods in the proposal will be described in Sect. 4.3. In the same way, those works that focus on comparing different algorithms will be discussed in Sect. 4.4.

**Fig. 15:**
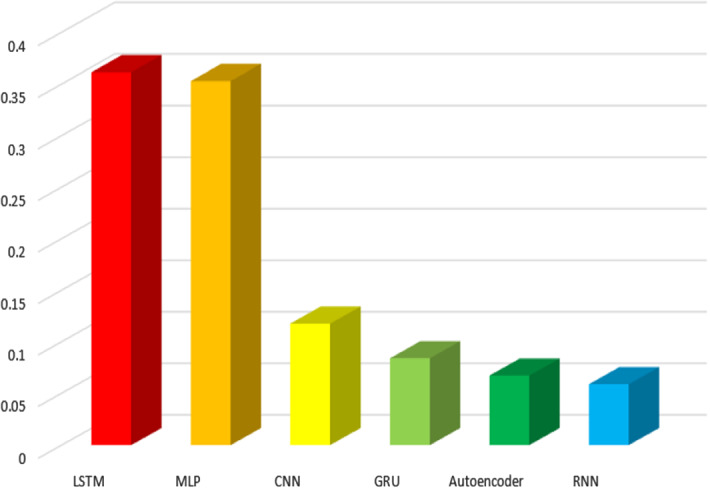
DL algorithms type proportion respect to the total number of DL algorithms

Figure 15 shows the different DL algorithms used in the analysed publications. LSTM networks and MLP networks are the most frequently used ($36.13\%$ and $35.3\%$ respect to all deep learning papers, respectively), followed by CNN. Next, we briefly describe the proposals which consider these algorithms.

Several papers apply MLP for predicting pollutant concentrations. In this group we can mention Paoli et al. (2011), focusing on $O_{3}$ in Corsica, Yao et al. (2012), predicting *PM*2.5 in Northern China, Huang et al. (2015), forecasting AQI in Shijiazhuang and Agarwal, Dedovic et al. (2016), predicting *PM*10 in Sarajevo, and Agarwal et al. (2020), considering *PM*10, *PM*2.5, $NO_{2}$ and $O_{3}$ in Delhi. In these proposals, the models are classical MLP neural networks without either significant improvements or variations. The highest number of hidden layers (15) corresponds to Dedovic et al. (2016). The highest number of predictor variables appears in Agarwal et al. (2020).

Other authors consider extensions and variations of the classical MLP model. Although the back-propagation algorithm is the most common training algorithm for neural networks, it can be improved as shown by Hoi et al. (2013). In order to make a prediction of the *PM*10 concentration in Macao, these authors use a Kalman filter in the learning algorithm to build a time-varying MLP neural network. Results show that the 3 layer time-varying MLP neural network is even more accurate than a 10 layer classical MLP neural network. Fu et al. (2015) developed an MLP network with rolling mechanism and accumulated generating operation of gray model to predict *PM*2.5 and *PM*10 concentrations in three Chinese cities. The original model was modified for improving its deficiency to assess the possible correlation between different input variables. Jiang and Li (2016) propose an MLP model for forecasting AQI in three Chinese cities using geo-sensor data using participatory sensing. Moreover, inspired by the dropout mechanism, the authors introduce a new approach in order to handle the problem of unsensed variables. A classical MLP improved by a linear regression technique is proposed by Thanavanich et al. (2021) to forecast *PM*2.5 in Thailand. Huang et al. (2020) introduce an improved MLP model employing Particle Swarm optimisation, a meta-heuristic evolutionary algorithm which simulates the behaviour of particles in nature. The target data is AQI in China.

LSTMs are also used in air quality forecasting models due to its effective performance in time series. Several works propose an LSTM-based model for prediction of different pollutants. Kök et al. (2017); Schürholz et al. (2019); Alhirmizy and Qader (2019); Li et al. (2020); Schürholz et al. (2020) apply LSTM to forecast AQI in Aarhus, Brasov, Australia, Madrid, and Melbourne, respectively. Alsaedi and Liyakathunisa (2019) and Jiao et al. (2019) present approaches to predict the level of AQI in Beijing and London and in Shanghai, respectively. Zhou et al. (2019) focus on the forecasting of *PM*2.5, *PM*10 and $NO_{X}$ in Taipei. Finally, Navares and Aznarte (2020) considered the levels of *CO*, $NO_{2}$, $O_{3}$, *PM*10, $SO_{2}$ and pollen in Madrid.

Many authors consider some variations of the classical LSTM model. Wang and Song (2018) introduce a three component algorithm to predict AQI in Beijing. The first component trains and combines dynamically several individual models that are based on weather patterns. The second component finds the spatial correlation between areas. The third component is an LSTM neural network. Song et al. (2019) propose an LSTM-Kalman model to predict three pollutants on experimental data-sets. Their results show that the proposed model has a higher accuracy than a classical LSTM model. Lan et al. (2020) propose an LSTM model to predict AQI in Hangzhou. The model presents a spatial optimisation component considering the correlations between variables and a temporal optimisation method considering the time window size. Their results also show the improvement of accuracy of the proposed model with respect to a classical LSTM. Seng et al. (2021) define a multi-index multi-output model based on LSTM to predict *PM*2.5, *CO*, $NO_{2}$, $O_{3}$ and $SO_{2}$ in Beijing. Han et al. (2021) train a Bayesian LSTM to predict AQI and *PM*2.5 in Beijing during a blue-sky period. Mao et al. (2021) proposed a temporal-sliding LSTM model to predict *PM*2.5 in the Jing-Jin-Ji region, in China. The model integrates the optimal time lag of spatio-temporal correlations to improve the model. Their results show that optimising the temporal-sliding LSTM reduces classical LSTM and MLP error. Jin et al. (2021) developed a nested LSTM network to forecast AQI in Beijing. Finally, Wang et al. (2021) propose an algorithm that uses a chi-square test to determine the most influential factors and an LSTM to forecast Shijiazhuang AQI. The results show that this model presents a smaller error than other classical methods.

Bidirectional LSTMs have been also considered in some works. The main difference between this algorithm and a classical LSTM is that the bidirectional LSTM preserves the past and future information, while the classical LSTM only preserves the past. This method is applied by Dua et al. (2019); Madaan et al. (2019) to predict *PM*2.5, *PM*10 and $NO_{2}$ in Delhi. Ma et al. (2019) consider a bidirectional LSTM and also apply transfer learning to transfer the knowledge learned from smaller temporal resolutions to larger temporal resolutions in order to forecast *PM*2.5, $NO_{2}$ and $O_{3}$ in Anhui, East of China. Ma et al. (2019) use a bidirectional LSTM combined with an inverse distance weighting technique for the spatio-temporal forecasting of *PM*2.5 in Guangdong. Ma et al. (2020) use a bidirectional LSTM, applying transfer learning, to predict *PM*2.5 also in Guangdong. Zhang et al. (2021) propose a bidirectional LSTM model, which includes an empirical mode decomposition, to predict *PM*2.5 in Beijing. Zhang et al. (2021) apply a bidirectional LSTM after they decompose the original time series with a variation mode decomposition method to predict *PM*2.5 in several Chinese cities.

Since LSTMs are considered an improvement of RNNs, that is the reason why RNNs are rarely used in air quality prediction. Ong et al. (2016) propose an RNN algorithm to predict *PM*2.5 in Japan. They employ a dynamic method to pre-train the model based on multi-step-ahead time series prediction. Lim et al. (2019) apply RNN to predict *PM*10, $O_{3}$, $SO_{2}$, *CO* and $NO_{2}$. In this case, the model corresponds to an RNN without significant improvements or variations with respect to the classical model.

GRUs are used in a similar proportion as RNNs, that is, scarcely. Zhang et al. (2020); Sonawani et al. (2021); Liu et al. (2021) propose enhanced GRU models for predicting AQI in the Huaihai Economic Zone, $NO_{2}$ in Pune (India) and *PM*2.5 in Beijing, respectively. The first two contributions apply a bidirectional GRU.

Finally, it is worth noting that a very low percentage of publications consider other algorithms. Multitask learning is a subfield of ML that improves the learning of a task by taking into account information of a related task. The idea is that what is learned for a task can help to better learn other tasks. A deep belief network with multi-tasking learning is employed by Li et al. (2019) to obtain a high level of accuracy. Sun et al. (2020) propose a model that uses multitask methods and RNN to forecast AQI in China. Barrón-Adame et al. (2012) propose a self organising map neural network to forecast $SO_{2}$ in Salamanca, Mexico. Wahid et al. (2013) forecast the $O_{3}$ concentration in Sydney considering a radial basis neural network. Fuzzy inference neural networks are used by Pai et al. (2013) to forecast oxidant concentration in Tokyo and by Ganesh et al. (2017) to forecast AQI in Delhi. Metia et al. (2014) consider a Matérn function based on a fractional Kalman filtering to forecast $O_{3}$ concentration in Sydney. De Vito et al. (2015) deal with a dynamic multivariate regression to forecast air quality in Cambridge. Some papers Liu et al. (2016); Zhao et al. (2016); Zhu et al. (2020); Sharma et al. (2020) introduce extreme learning machine models to forecast AQI in Beijing, air quality in Helsinki metropolitan area, AQI in Liaoning (China) and particulates matter in four Australian cities, respectively. Mei et al. (2016) develop an Elman neural network model using a genetic algorithm (GA) to optimise the initial weights and the number of hidden layer nodes. This model is applied to forecast AQI from a public data-set chosen from the UCI ML repository. A wavelet neural network is proposed by Zhang et al. (2018) to make a short-term forecasting of AQI in Beijing. An air pollution forecasting system which uses deep distributed fusion network is proposed by Yi et al. (2018), providing fine-grained AQI forecasts for more than 300 Chinese cities. Li et al. (2018) consider data spatial-temporal features to propose a spatio-temporal aware sparse denoising auto-encoder to forecast AQI in China. Their results show that use of spatial-temporal features increases accuracy. Yan et al. (2018) use an EDNN to predict *PM*2.5 in Tianjin. Wu and Zhang (2019) propose a generative additive model to forecast *PM*2.5 in Beijing. A leveraging bagging ensemble learning algorithm is proposed by Liu et al. (2019) to predict the air quality grade in Beijing. Finally, Yi et al. (2020) apply a distributed fusion network for short-term predictions and a deep distributed cascade network for long-term predictions to forecast the value of AQI of the next 48 h and the daily average air quality of the next 7 days.

We think that the popular use of LSTM is due to its high adaptability to solve sequential problems in general and time series problems in particular. In the case of MLP, we think that they are very often used due to is popularity, although its usage in time series problems can present some difficulties. CNN, GRU and Auto-encoders are algorithms whose main application field is very different to time series. We think that this is the cause of their low use. Finally, the popularity of RNNs has decreased in recent years because LSTM improves their performance.

### Regression algorithms

In this section we consider contributions that apply a unique regression algorithm. Approaches that combine different regression methods in the proposal will be described in Sect. 4.3. Likewise, those papers that compare different algorithms will be discussed in Sect. 4.4.

**Fig. 16:**
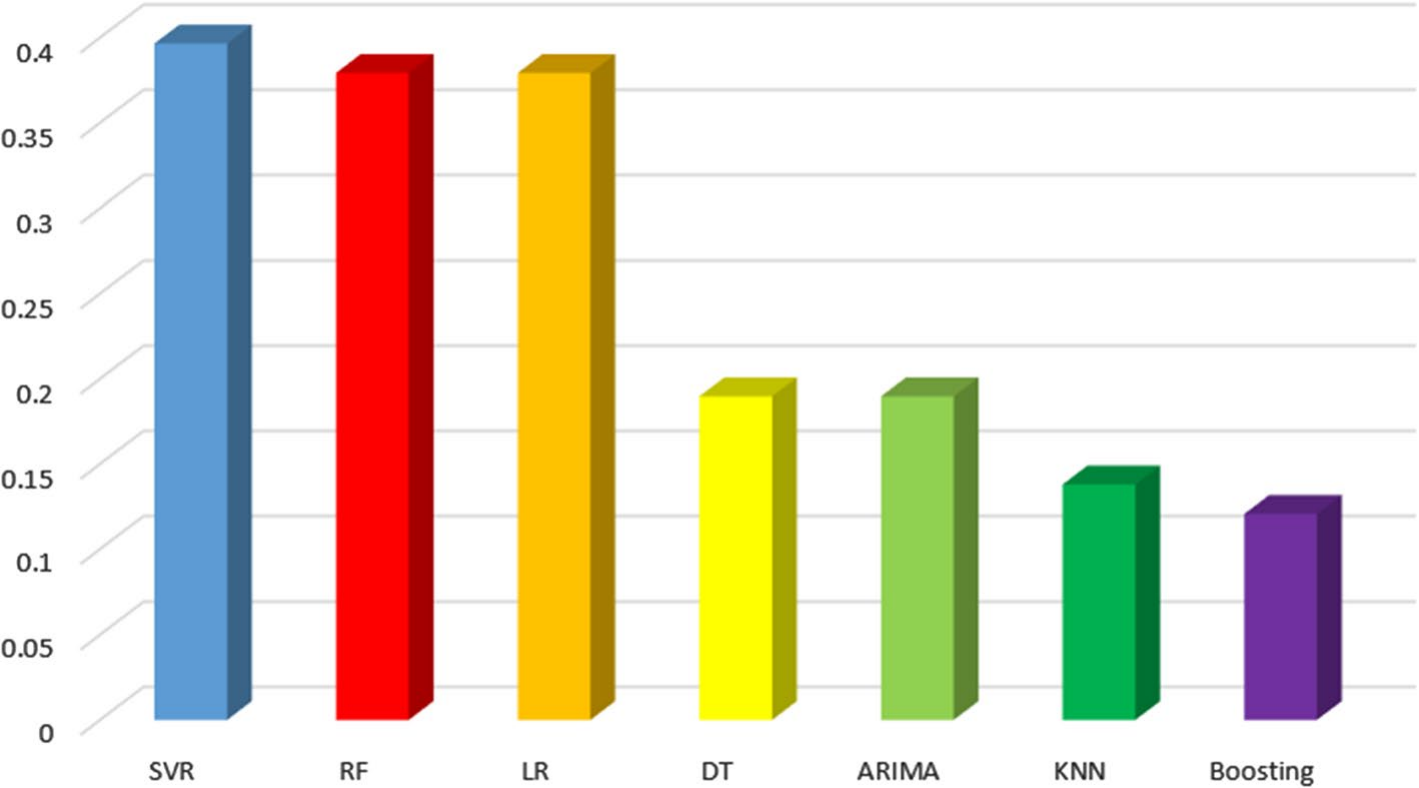
Regression algorithms type proportion respect to the total number of regression algorithms

Figure 16 shows the percentage of analysed publications that deal with the different regression algorithms. We can see that SVR, RF and linear regression are the most used methods in the analysed works. Note that the percentages add up to more than 1. The reason for this is that many papers introduce more than a single regression algorithm.

An SVR model with Gaussian and polynomial kernel is proposed by Sotomayor-Olmedo et al. (2011) to forecast *PM*2.5 and *PM*10 in London. A SVR model is developed by García Nieto et al. (2013) to built a non-dynamic forecasting model of AQI in Oviedo, Spain. SVR with quasi-linear kernel is designed by Zhu and Hu (2019) to predict *CO*, $NO_{X}$ and $NO_{2}$ in an open data-set. In this case, their results show that quasi-linear SVR outperforms other learning methods.

Zhang et al. (2015) develop a model, in Apache Spark, using a parallelised RF algorithm to forecast *PM*2.5 in Beijing. The model takes into account 12 predictor variables and the results show that their improvements imply high accuracy in the model. Li et al. (2018) propose an algorithm to predict *PM*2.5, $NO_{2}$ and $SO_{2}$ in Beijing. They apply a method based on an RF for the selection of the most relevant factors. Initially, they obtain 20 predictor variables for forecasting the concentration of the pollutants but some of them are removed by using a selection method based also on an RF.

Linear regression (simple or multiple) is the most used *classical* regression algorithm in the literature. Barthwal and Acharya (2018) propose a linear regression model to predict AQI levels in New Delhi by taking into account 8 predictor variables.

Jato-Espino et al. (2018) use clustering to partition the air quality monitoring stations according to similarity characteristic. Then, they apply multi-linear regression to predict AQI in Catalonia. Durić and Vujović (2020) apply multi-linear regression, using 3 predictor variables, to forecast AQI in four stations of Belgrade.

A similar model is applied by Ma et al. (2020) for predicting AQI in North China using SPSS program. They use 7 predictor variables and obtain a highly accurate model. LASSO, a multi-linear regression regularise method for variable selection is used by Sethi et al. (2021) to predict AQI in Delhi.

Some works deal with ARIMA algorithms. Liu et al. (2018) use an ARIMA algorithm combined with numerical modelling to forecast *PM*2.5, $O_{3}$ and $NO_{2}$ in Hong Kong. The effectiveness of this combination is shown by empirical results. An ARIMA model is developed by Ngom et al. (2021) to forecast *PM*10 with a multi-agent real-time simulation in Dakar, Senegal. They also discuss the relevance of studying other parameters of the model. A seasonal ARIMA method is developed by Bhatti et al. (2021) to predict *PM*2.5 in Lahore, Pakistan. Hajmohammadi and Heydecker (2021) propose a vector auto-regressive moving-average model to predict *PM*10, *NO*, $NO_{X}$ and $NO_{2}$ in London.

Another works consider the use of other regression algorithms. A method which uses rolling window regression is developed by Lang et al. (2019) to estimate trends in $NO_{2}, NO_{X}$ and $NO_{2}/NO_{X}$ in London. Wang and Kong (2019) develop a DT to predict AQI for different locations in China. The model is improved in the feature attribute value and in the weighting of the information gain. Results show that this model provides best accuracy than other improved DT models. Zhang et al. (2019) propose an improved light gradient boosting machine method to predict *PM*2.5 in Beijing and Matović and Vlahović (2021) propose a boosting algorithm to forecast 6 pollutants in Beijing.

We think that the popular use of SVR and RF is due to its great adaptability to different contexts and to different type of variables (this is specially remarkable in the case of RF). In the case of LR, its significant percentage of use can be due to being the basis of regression algorithms. DT is scarcely used because RF provides a clear improvement and, with the same knowledge, one can apply a DT or an RF indistinctly. In the case of ARIMA, we think that it is not used very often because its performance decreases when many predictor variables are included, as in the cases that concern us. Finally, KNN and Boosting are more popular to solve classification tasks than regression tasks.

### Hybrid models

In this section, we will review papers that consider different algorithms in their proposals.

First, we review hybrid models that only deal with deep learning algorithms. Zheng et al. (2013) developed a co-training model to forecast the *PM*2.5 concentration in Beijing and Shanghai. The proposed model is a linear-chain conditional random field model used to model the temporal predictor variables and an MLP network with one hidden layer which takes spatially-related predictor variables as input. Lin et al. (2018) present a geo-context based diffusion convolutional recurrent neural network, a hybrid CNN-RNN model, for *PM*2.5 short-term forecasting in Los Angeles and Beijing. The combination of an EDNN and a GRU results in a model based on *n*-step recurrent prediction. This model, named *seq-2-seq*, is applied by Liu et al. (2019) to forecast AQI in Beijing. Zhang et al. (2020) propose a hybrid CNN-GRU multi-task learning model to predict *PM*2.5 in three districts of Lanzhou, China. Hu et al. (2021) combine an MLP network with a linear interpolation method to forecast *PM*2.5 and *PM*10 in two Chinese cities. A hybrid EDNN-LSTM model was developed by Abirami and Chitra (2021) to predict AQI in Delhi. In this case, the EDNN encodes the spatial relations in data. A hybrid model based on a general regression neural network, a extreme learning machine and a variational mode decomposition, was proposed by Rahimpour et al. (2021) to predict AQI in Urmia, Iran.

The combination of LSTM and CNN increases the model efficiency due to both the LSTM long-term forecasting capability and the CNN capability to learn data features without the need of extracting them in advance. Hybrid models based on this combination are developed by Zhao et al. (2019); Li et al. (2020); Kiftiyani et al. (2021); Jovova and Trivodaliev (2021); Le et al. (2020); Putra et al. (2021) to predict AQI in the Beijing-Tianjin-Hebei region, *PM*2.5 in Beijing, 6 pollutant concentrations in Seoul, *PM*10 in Skopje, AQI in Seoul and *PM*2.5 in Taiwan, respectively. Previously, Soh et al. (2018) apply a similar hybridisation in which an MLP is also combined with a CNN and a spatio-temporal LSTM. A hybrid model which combines LSTM, GRU and CNN is developed by Chiang and Horng (2021) to forecast daily *PM*2.5 in Taiwan.

As we previously said, some proposals consider the use of GAs to determine the optimal initial weights. Zhenghua and Zhihui (2017) apply a hybrid GA-MLP model to predict AQI in Xuchang. Kang and Qu (2017) and Su and Xie (2020) apply this hybridisation to forecast AQI in Lanzhou and Dailan, respectively. The results show that GA optimisation reduces the model error with respect to a non-optimise MLP. Mu et al. (2017) and Yang et al. (2020) propose a similar algorithm to predict AQI in Taicang and Shanghai, respectively. In these works, principal component analysis (PCA) is used to reduce dimension before the application of the algorithm and shows its effectiveness for increasing the accuracy. Lin et al. (2020) develop a hybrid approach which includes a self-organising-maps neural network, a GA for optimisation and PCA for reducing the dimension of a neuro-fuzzy modelling, a combination of a neural network and fuzzy logic, to predict AQI in Taiwan.

Some publications propose other combinations of deep learning algorithms. Ao et al. (2019) use a bidirectional LSTM combined with a k-means clustering to forecast AQI in Qingdao. A Bayesian encoder-decoder hybrid RNN is developed by Han et al. (2020) to forecast *PM*2.5 and *PM*10 in London and Beijing. Zhao et al. (2020) propose a hybrid model which combines the advantages of both forward neural networks and recurrent neural networks to forecast AQI in Lanzhou and Xi’an. A hybridisation of GA and the Encoder-decoder method is developed by Nguyen et al. (2021) to predict *PM*2.5 concentrations in Hanoi and Taiwan. (Ouyang et al. 2021) combine a dynamic graph CNN with a GRU to forecast *PM*2.5 concentration in two datasets belonging to Beijing and London. A hybridisation between a CNN with residual connections, attention layers and a bidirectional LSTM is developed by Choi et al. (2021) to forecast *PM*2.5 and *PM*10 in Seoul. Colchado et al. (2021) propose a model composed by a KNN-attention pooling layer stacked to a classical MLP network to make a spatial prediction of *PM*2.5 in São Paulo.

Finally, we will review those papers which only consider the hybridisation of regression algorithms. Yi et al. (2019) propose a hybrid boosting-random forest model to classify AQI in Fangchenggang and Beijing. The model uses samples grouped bootstrap instead of a single bootstrap method in the random sampling phase. Results show that this improvement provides best accuracy than an RF method. Gocheva-Ilieva et al. (2020) propose a hybrid model that combines RF and ARIMA: RF is used to analyse meteorological feature relations and ARIMA is used to correct the model. The model is applied to forecast the concentration of 7 pollutants in Dimitrovgrad, Bulgaria. Hybrid models based on the combination of ARIMA, optimised extreme learning machine and fuzzy time series models are developed by Li et al. (2020) to forecast AQI in Shijiazhuang, Zhengzhou and Guangzhou.

Some works also propose hybridisation of deep learning and regression algorithms. Bougoudis et al. (2016) propose a hybrid machine learning system which combines RF, self-organising maps and fuzzy inference systems that has been applied to forecast AQI in Athens. Chen et al. (2017) propose a hybrid model which combines an SVR and an Elman neural network to predict *PM*2.5 in Wuhan. Wang et al. (2017) present a hybrid model where they optimise the parameters using four probabilistic parameter models. Then, an improved MLP neural network with signal processing techniques is employed to predict AQI in several Chinese cities. Qi et al. (2018) introduce a hybrid model which combines an ECNN and a spatio-temporal semi-supervised regression model to forecast fine-grained AQI in Beijing. An ERT-MLP hybrid model is developed by Eslami et al. (2019) to forecast $O_{3}$ concentration in Seoul. Zhu et al. (2020) propose a model which combines a complementary ensemble empirical mode decomposition to pre-process data and statistical machine learning models to forecast AQI in 17 port cities in China. An unsupervised model is proposed by Guo et al. (2020) to predict *PM*2.5 in Hubei, China. The model corresponds to a hybrid of a GRU and an inverse distance weighted KNN. GRU is also combined with multi-linear regression by Lin et al. (2021) and applied to forecast *PM*2.5 concentration in Taiwan. Janarthanan et al. (2021) propose a hybridisation of SVR and LSTM to predict AQI in Chennai.

### Papers comparing different models

**Fig. 17:**
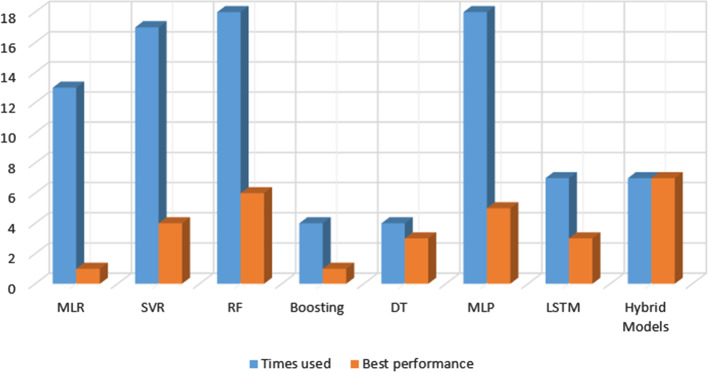
Algorithm type used in papers comparing models and number of times showing the best performance

In this section we review papers that compare different approaches with the goal of showing which one of them outperforms the others under different scenarios. These papers represent $23.5\%$ of all the papers analysed in our work. It is worth pointing out that most of these works focus on DL algorithms because they outperform regression algorithms more than 60% of the cases when both types of algorithms are compared. Figure 17 summarises the number of times that each model is used and when these models show better results than the considered alternatives.

Some works show that LSTM is the most accurate algorithm when it is compared with some deep learning and regression algorithms. Thaweephol and Wiwatwattana (2019) compare a usual LSTM model and a seasonal ARIMA with exogenous regressor for *PM*2.5 forecasting in the Bangkok urban area. The experiments indicate that LSTM has a low level of error for each of the time steps. Chowdhury et al. (2020) compare several models (DT, RF, SVM, Bagging, MLP or LSTM) to forecast AQI in the city of Dhaka, Bangladesh. Results show that LSTM models present the optimal performance when predicting hourly and daily values. Zhoul et al. (2020) compare a hybrid prophet-LSTM model with a hybrid prophet-SVM model to forecast AQI in Nanjing from 2014 to 2019. The results of the performed experiments show that hybrid prophet-LSTM presents the best performance and the highest accuracy. Cheng and Peng (2021) develop a hybrid model which combines an LSTM network with a fuzzy algorithm to forecast AQI in Taiwan.

Other publications conclude that MLP is the most accurate algorithm. Five predictive models (SVR, Naive-Bayes, RF, MLP and KNN) were compared by Amado and Dela Cruz (2018) to forecast AQI in China. All the models obtained an accuracy higher than $94\%$, corresponding the highest one to the MLP model. Abdullah et al. (2019) compare an MLP model with an MLR to forecast *PM*10 in Kuala Lumpur, Malaysia. Results show that the highest $R^2$ is obtained when using MLP. Several regressor models (MLR, KNN, RF, DT or MLP) were compared by Maheshwari and Lamba (2019) to predict *PM*2.5 in Beijing. Although the performance of all the models was comparable, MLP achieved the highest true positive rate with an accuracy value of 95.4. Bouakline et al. (2020) compare three models (SVR, MLP and RF) to forecast *PM*10 in Casablanca, Morocco. Again, their results show that MLP has the best accuracy.

In the same way, there are works that compare several models and conclude that RF is the most accurate algorithm. Ochando et al. (2015) compare several algorithms (MLR, KNN, M5P, RF, SVM or MLP) to predict different pollutant concentrations in Valencia, Spain. RF obtained the highest accuracy. Contreras and Ferri (2016) compare five algorithms (MLR, quantile regression, KNN, M5P -a decision tree learner for regression task- and RF) to forecast the level of several pollutants also in Valencia. In this case, RF also reached the highest accuracy. Shawabkeh et al. (2018) compare an MLP algorithm with an SVR model to forecast the concentration of benzene in a city of Italy. MLP obtains the best results both in short-term and long-term cases. Ameer et al. (2019) make a comparison between four models (RF, DT, MLP, Boosting) to predict *PM*2.5 of several Chinese cities. RF obtains the best accuracy. Li et al. (2019) make a comparison between logistic regression and RF to forecast AQI in California. The best performance was exhibited by the RF model. Srikamdee and Onpans (2019) compare three different methods (MLR, MLP and genetic programming) to forecast AQI in several areas of Thailand. Somehow surprisingly, the MLR and the genetic programming approach obtain the best performance in clean and unhealthy air quality situations. Kuo et al. (2019) develop a comparison to predict AQI in Taipei during one year. This paper compare an MLP, an RNN and an RNN with a Gaussian process. The last one obtains the highest accuracy. Mahanta et al. (2019) compare the accuracy of some prediction models (original MLR, two MLR variants, MLP, RF, extra trees, boosting and KNN) to forecast AQI in New Delhi. The results show that the extra trees regression model obtains the highest accuracy. Pasupuleti et al. (2020) consider three algorithms (RF, DT, MLR) to forecast the concentration of several pollutants in Spain. Various regressor methods (MLP, RF, DT and SVR) are compared by Kaur Bamrah et al. (2020) for predicting AQI in India by adopting terrain features. In these last two papers, RF obtains the highest accuracy. Zheng et al. (2020) compare several regression and DL models (ARIMA, LSTM, SVR, RF, MLR or several boosting models) to forecast AQI in Beijing and Hong Kong. Results conclude that ensemble models outperform the accuracy of individual models. Eight regression models are compared by Hota et al. (2021) to forecast AQI in Indian cities during the COVID-19 crisis. Results show that boosting models obtain the best results. Yarragunta et al. (2021) compare six regression algorithms (DT, KNN, SVR, MLR, RF and Naive-Bayes) to predict AQI in Delhi. In this case, the highest accuracy is obtained by the DT algorithm. Chakradhar Reddy et al. (2021) develop a comparison between six supervised ML models (LR, RF, DT, SVR, KNN and Naive-Bayes) to forecast AQI in New Delhi. Results show that DT obtains the higher accuracy (very close to $100\%$).

There are several papers where SVM obtains the best results when it is compared with other algorithms. In the comparison developed by Ganesh et al. (2017) between four regression models to forecast AQI in India and USA, SVM with Gaussian kernel gets a maximum $R^2$ value. The algorithms are evaluated in two different cities, which provides a high consistency. Srivastava et al. (2018) compare eight ML models and the best $R^2$ value is obtained by the SVR and MLP models. However, the results are substantially worse than the ones of the previous paper. Mahalingam et al. (2019) compare an SVR with an MLP, getting the former the best accuracy ($97.3 \%$). Gu et al. (2020) compare an SVR algorithm that has been optimised by using PSO with an SVR algorithm that has been combined with an improved PSO algorithm to forecast AQI in Shenzhen. Results show that the improved algorithm requires less execution time and has higher accuracy. Sunori et al. (2021) compare a regression model (DT) and a DL model (general regression neural network) to forecast *PM*10 in Uttarakhand, India. The experiments show that the regression model has better results. Verma et al. (2021) also compare a ML model (ARIMA) and a DL model (LSTM) to forecast AQI in Delhi. In this case, LSTM outperforms ARIMA in three different metrics.

Several studies show that hybrid models perform usually better than single models. Metia et al. (2016) compare the performance of an extended fractional Kalman filter and an extended Kalman filter to predict two pollutant emissions in Sidney. The results show that the first model obtains the lowest error. An Elman RNN, a Jordan RNN and a hybrid Elman-Jordan RNN are compared by Septiawan and Endah (2018) to forecast several pollutant concentrations in London. The results show that Jordan RNN obtains the lowest RMSE. Ali Shah et al. (2019) compare a phase-space reconstruction algorithm combined with an RF, with an SVM and with an MLP neural network to forecast air quality in Masfalah, Saudi Arabia. The combination with the MLP network obtains the best results. Four prediction models [ARIMA, principal component regression (PCR), ARIMA-PCR hybridisation and combination of ARIMA and Gene Expression Programming (GCP)] are compared by Shishegaran et al. (2020) to forecast daily air quality in Tehran. The ARIMA-GCP model obtains the lowest error by far. Ozturk (2021) compares a hybrid model which combines RF, SVR and radial basis function regressor with an LSTM and a GRU to forecast *CO* and $NO_{2}$ in a provided dataset. Results show that the hybrid model obtains the lowest error. To forecast AQI in Beijing, Yan et al. (2021) compare four models (CNN, LSTM, hybrid CNN-LSTM and an spatio-temporal model). The overall forecasting based on the LSTM is considered as the optimal model for all stations.

We conclude this section with those algorithms that have been included in a lower percentage of papers where different approaches are compared. Campalani et al. (2011) compare three co-kriging models (2-variate ordinary co-kriging, 2-variate universal co-kriging and 4-variate ordinary co-kriging) to estimate *PM*10 concentration in Emilia Romagna, Italy. Their results show that the 2-variate ordinary co-kriging obtained the lowest error. This is the only analysed work which employs co-kriging methods to forecast. Asadollahfardi et al. (2016) make a comparison between two different neural networks (MLP and RBNN) to forecast *PM*2.5 in Karaj, Iran. In this case, RBNN achieves the maximum $R^2$ value. MLR has showed the best results in Karatzas et al. (2018), where the authors compare an MLP, an RF and an MLR to *PM*10 in Gdansk, Poland, and Athens. Multi-linear regression obtained the lowest level of error. Radial-basis neural networks show the best performance in two works. Yadav and Nath (2018) compare a RBNN and a generalised regression neural network to forecast *PM*10 in India. The obtained results show that RBNN minimises the error.

## Conclusions

The aim of this paper is to give a survey including the approaches presented on air quality forecasting since 2011. In order to select papers, we searched a previously defined query in the most widely used scientific databases: *ACM Library*, *Elsevier Online Library*, *IEEE Xplore*, *Springer-Link* and *Wiley Online Library*. After removing papers not related to the main topic of the survey, 155 publications were selected. Then, several characteristics of the papers were extracted and analysed.

In geographic terms, the analysed papers show a direct correlation between the most polluted and the most studied countries.

In temporal terms, there is a clearly increasing trend in the number of papers published per year. This is mainly due to the popularisation of ML in recent times and to the increase of awareness about problem of pollution. However, this trend changes in 2021. We have evidence to think that this change is due to the fact that many researchers are using COVID-19 data to validate their models.

Concerning the studied pollutant measures, our analysis shows that AQI is used in approximately half of the studied papers. Concerning pollutant concentrations, *PM*2.5 is the most predicted, in a total of 54 papers. The hazard of this pollutant could be the main reason of that.

We have observed that pollutants features are the type most widely used (around $50\%$ of the papers). Weather variables are also used very often (around $40\%$ of the papers).

Focusing on ML techniques, the results show that DL algorithms are most popular than regression algorithms. Furthermore, some hybrid algorithms include both types of algorithms in the same model. The DL algorithms most used are LSTM and MLP ($27.6\%$ and $26.9\%$ of the total respectively). Other DL algorithms less frequently used are CNN, RNN, GRU and auto-encoders. Regression algorithms most used are SVR ($14.7\%$ of the total) and RF ($14.1\%$ of the papers). Other regression algorithms less frequently used are DT, ARIMA, KNN and Boosting.

Finally, we would like to look beyond the current work. We can mention recent work that give us an idea of the new trends in the application of Machine Learning to forecast air quality. A comprehensive survey on the correlation between air quality and climate change to develop techniques and models that enhance early warning mechanisms and support an effective response to climate-change-induced air pollution, thereby fostering sustainable cities and societies, has been recently presented Balogun et al. (2021). Concerning trends on new algorithms, we must mention the use of Deep Transformer Networks. Although they were initially developed to solve natural language processing tasks, their use has been extended in recent years to other fields such as time series and, by extension, to air quality forecasting. Transformers have been recently used to forecast *PM*2.5 concentration in two Chinese cities (Zhang et al. 2022) and ozone concentration in Madrid, Spain Méndez et al. (2022). Another interesting family of models which are recently increasing their popularity in air quality forecasting are Graph Neural Networks. The main characteristic of this type of networks is the information obtained from the dynamic interaction between the neighbours (e.g. different cities, different neighbourhoods, different streets) that are weighted depending on the distance. In this line, a recent work Li et al. (2021) has applied a Graph Neural Network in three real-world datasets to predict *PM*2.5 concentration, obtaining better performance than baseline models. We can also mention the recent application of Temporal Convolutional Networks (TCNs) to air quality prediction, in particular, to predict *PM*2.5 concentrations. In this line, a multi-output TCN was used to predict *PM*2.5 and *PM*10 concentrations in multiple sites Samal et al. (2021), a multi-directional TCN was proposed to forecast *PM*2.5 in a dataset with missing values Samal et al. (2021), and a Gaussian-TCN model was recently used to predict *CO* concentration Ni et al. (2023). Finally, it is worth to mention the recent application of Complex Event Processing (CEP) Corral-Plaza et al. (2021) to analyse and predict air quality Semlali et al. (2021).

## Footnotes

1 https://www.scmp.com/news/world/europe/article/3149735/air-pollution-kills-7-million-year-says-who-it-tightens, last accessed on 2022-02-15.

2 https://www.dataversity.net/a-brief-history-of-machine-learning/, last accessed on 2022-02-10.

3 https://ourworldindata.org/, last accessed on 2022-02-15.

4 https://www.iqair.com/, last accessed on 2022-02-15.

5 https://www.airqualitynow.eu/about_indices_definition.php, last accessed on 2022-02-15.

6 https://datadrivenlab.org/air-quality-2/chinas-new-air-quality-index-how-does-it-measure-up/, last accessed on 2022-02-15.

7 https://www.airnow.gov/aqi/aqi-basics/, last accessed on 2022-02-15.

8 https://web.archive.org/web/20111115230340/http://www.comeap.org.uk/images/stories/Documents/Reports/comeap%20review%20of%20the%20uk%20air%20quality%20index.pdf, last accessed on 2022-02-15.

9 https://www.eea.europa.eu, last accessed on 2022-02-15.

10 https://www.who.int/, last accessed on 2022-02-15.

11 https://www.comeap.org.uk, last accessed on 2022-02-15.
